# Supplementary material for: Drug repurposing for aging research using model organisms
Source: Aging Cell. 2017 Jun 16;16(5):1006–15. doi: 10.1111/acel.12626 (PMC5595691; doi:10.1111/acel.12626)
Supplement: Supplementary file 7 — Data S1 Zip‐Archive of all report cards. [file ACEL-16-1006-s007.zip › RC_6CM.pdf]

6CM

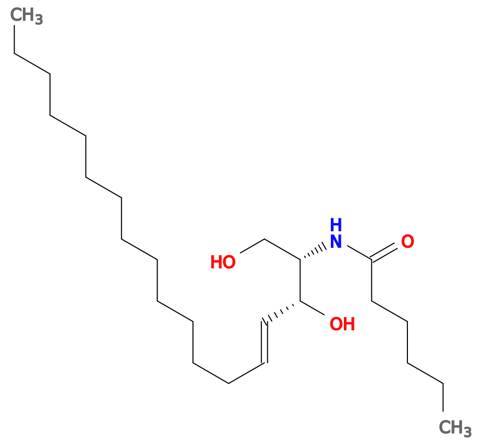

Database identifiers

ChEMBLCompound	CHEMBL224877
CHEBI	63867
ZINC	ZINC16051948
eMolecules	716521

Ranking

	Rank	Score
Drosophila	467/697	0.307
C. elegans	453/591	0.11

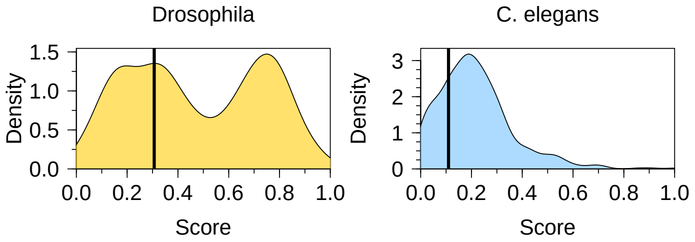

	Ageing implication	Domain conservation	Binding site conservation	Binding affinity	Bioavailability	Lipinski	Promiscuity	Purchasability	Drug approval	**Total**
Drosophila	0.36	0.897	0.938	0.943	(0.9)	-0.05	-0.0	0.1	0.0	0.307
C. elegans	0.36	0.87	0.916	0.943	0.222	-0.05	-0.0	0.1	0.0	0.11

Names

- **N-(hexanoyl)sphing-4-enine**
- (2S
- 3R,4E)-Ceramide
- C6-Cer
- C6-ceramide
- Cer(d18:1/6:0)
- D-erythro-N-hexanoylsphingosine
- N-hexanoyl-D-erythro-sphingosine
- N-Hexanoylspingosine

Roles

ChEBI entry 63867 has no roles

Status

Approved drug (according to ChEMBL)	No
Number of Rule of 5 violations	1
Binding affinity to original target in log units (RF-Score prediction)	7.81
Burns *C. elegans* bioavailability prediction	-4.02

Compound Target Characteristics

Collagen type IV alpha-3-binding protein

Best gene implication in ageing for this target family came from gene Q9Y128 via mapping the annotation from Ensembl FBgn0027569 via mapping the annotation from EntrezGene 38928 via mapping the annotation from GenAgeModels 0117 annotated in GenAge release 17.

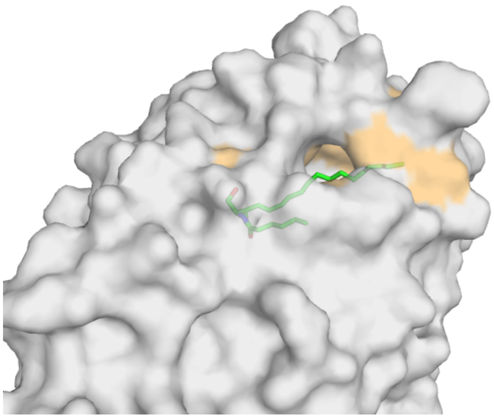

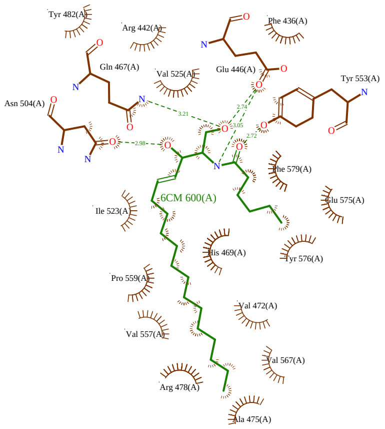

protein	amino acids contacts (binding site)																		
PDB:2e3n:chainA:Q9Y5P4	F	R	E	Q	H	V	A	R	Y	N	I	V	Y	V	P	V	E	Y	F
sp:Q9Y5P4:C43BP_HUMAN	F	R	E	Q	H	V	A	R	Y	N	I	V	Y	V	P	V	E	Y	F
tr:D4ACN6:D4ACN6_RAT	F	R	E	Q	H	V	A	R	Y	N	I	I	Y	V	P	V	E	Y	F
sp:Q9EQG9:C43BP_MOUSE	F	R	E	Q	H	V	A	R	Y	N	I	I	Y	V	P	V	E	Y	F
tr:Q9Y128:Q9Y128_DROME	F	R	E	Q	H	I	A	R	F	N	L	V	Y	V	P	A	E	Y	F
tr:Q93569:Q93569_CAEEL	F	K	D	Q	H	V	A	R	F	N	L	V	Y	V	P	A	E	Y	F

	whole protein		domain-based		contact-based	
protein	ident	simil	ident	simil	ident	simil
PDB:2e3n:chainA:Q9Y5P4	0.99	1.0	0.99	1.0	1.0	1.0
sp:Q9Y5P4:C43BP_HUMAN	1.0	1.0	1.0	1.0	1.0	1.0
tr:D4ACN6:D4ACN6_RAT	0.96	0.99	0.98	1.0	0.95	0.98
sp:Q9EQG9:C43BP_MOUSE	0.97	0.99	0.98	1.0	0.95	0.98
tr:Q9Y128:Q9Y128_DROME	0.42	0.74	0.47	0.82	0.79	0.94
tr:Q93569:Q93569_CAEEL	0.24	0.61	0.37	0.79	0.74	0.92

cert (FBgn0027569) associated phenotypes

heat stress response defective, locomotor behavior defective, short lived, stress response defective

(Information from FlyBase)
